# Out-of-pocket fertility preservation expenses: data from a Japanese nationwide multicenter survey

**DOI:** 10.1007/s10147-024-02614-z

**Published:** 2024-09-04

**Authors:** Masanori Ono, Yasushi Takai, Miyuki Harada, Akihito Horie, Yidan Dai, Eiji Kikuchi, Mitsuru Miyachi, Tetsuya Yamamoto, Nobuharu Fujii, Hiroaki Kajiyama, Atsushi Manabe, Toshiaki Yasuoka, Shinji Katsuragi, Keiko Mekaru, Tadashi Maezawa, Yuki Horage, Shinsuke Kataoka, Robert Nakayama, Takako Eguchi Nakajima, Fuminori Kimura, Chikako Shimizu, Kohei Sugimoto, Seido Takae, Yasushi Yumura, Hirotaka Nishi, Tatsuro Furui, Ken-Ichirou Morishige, Chie Watanabe, Yutaka Osuga, Nao Suzuki

**Affiliations:** 1 Department of Obstetrics and Gynecology, Tokyo Medical University, 6-7-1 Nishi Shinjuku, Shinjuku-ku, Tokyo, 160-0023 Japan; 2 Department of Obstetrics and Gynecology, Saitama Medical Center, Saitama Medical University, 1981 Kamoda, Kawagoe-shi, Saitama, 350-8550 Japan; 3 Department of Obstetrics and Gynecology, Graduate School of Medicine, The University of Tokyo, 7-3-1 Hongo, Bunkyo-ku, Tokyo, 113-0033 Japan; 4 Department of Obstetrics and Gynecology, Medical Research Institute KITANO HOSPITAL, PIIF Tazuke-Kofukai, 2-4-20 Ohgimachi, Kita-ku, Osaka, 530-8480 Japan; 5 Department of Urology, St. Marianna University School of Medicine, 2-16-1 Sugao, Miyamae-ku, Kawasaki, Kanagawa 216-8511 Japan; 6 Department of Pediatrics, Graduate School of Medical Science, Kyoto Prefectural University of Medicine, Kyoto, 602-8566 Japan; 7 Department of Neurosurgery, Yokohama City University Graduate School of Medicine, Yokohama, Japan; 8 Division of Transfusion and Cell Therapy, Okayama University Hospital, Okayama, Japan; 9 Department of Obstetrics and Gynecology, Nagoya University Graduate School of Medicine, Nagoya, Japan; 10 Department of Pediatrics, Hokkaido University, Sapporo, Japan; 11 Department of Obstetrics and Gynecology, Graduate School of Medicine, Ehime University, Ehime, Japan; 12 Department of Obstetrics and Gynecology, Faculty of Medicine, University of Miyazaki, Miyazaki, Miyazaki Japan; 13 Department of Obstetrics and Gynecology, Graduate School of Medicine, University of the Ryukyus, 207 Uehara Nishihara, Okinawa, 903-0215 Japan; 14 Department of Obstetrics and Gynecology, Mie University School of Medicine, Mie, Japan; 15 Department of Obstetrics and Gynecology St, Marianna University School of Medicine, Kawasaki, Japan; 16 Department of Pediatrics, Nagoya University Graduate School of Medicine, Nagoya, Japan; 17 Department of Orthopedic Surgery, Keio University School of Medicine, Tokyo, Japan; 18 Department of Early Clinical Development, Kyoto University Graduate School of Medicine, Kyoto, Japan; 19 Department of Obstetrics and Gynecology, Nara Medical University, 840 Shijo-cho, Kashihara, 634-8522 Japan; 20 Department of Breast and Medical Oncology, National Center for Global Health and Medicine, 1-21-1 Toyama, Shinjuku-ku, Tokyo, 162-8655 Japan; 21 Center for Reproductive Medicine, Dokkyo Medical University, Saitama Medical Center, 2-1-50 Minamikoshigaya, Koshigaya, Saitama 343-0845 Japan; 22 Reproduction Center, Medical Center, Yokohama City University, Urafune Cho 4-57, Minami-ku, Yokohama, Kanagawa 232-0024 Japan; 23 Department of Obstetrics and Gynecology, Gifu University Graduate School of Medicine, 1-1 Yanagido, Gifu-shi, Gifu, 501-1194 Japan; 24 Department of Obstetrics and Gynecology, Osaka General Medical Center, 3-1-56 Mandaihigashi, Sumiyoshi-ku, Osaka, 558-8558 Japan; 25 Department of Nursing School of Nursing and Rehabilitation Sciences, Showa University, 1-5-8 Hatanodai Shinagawa-ku, Tokyo, 142-8555 Japan

**Keywords:** Assisted reproductive technology, Adolescent and young adult, Cancer, Fertility preservation, Out-of-pocket fertility preservation expenses, Reproduction

## Abstract

**Background:**

The expenses related to fertility preservation or subsequent assisted reproductive treatments are significant for adolescents and young adult patients in Japan’s current healthcare system. With fertility preservation becoming more widespread in developed countries, it is expected that these costs will be covered by insurance or subsidies. It is critical for patients, healthcare providers, and the government to know the costs that patients will be responsible for. In Japan, the costs of fertility preservation and subsequent assisted reproductive technology are not covered by insurance, but patients can apply for subsidies from the local and central governments if certain conditions are met. Presently, the above-mentioned costs, as well as the amount paid by the patient, vary by facility. Therefore, it is essential to ensure patients’ continued access to necessary medical care despite the associated costs.

**Methods:**

In this study, questionnaires were mailed to 186 certified fertility preservation facilities in Japan to assess patients who had undergone fertility preservation or assisted reproduction. The questionnaires were sent between October 27, 2023 and March 31, 2024, with 140 of the 186 facilities responding (response rate: 75.3%).

**Results:**

Our findings show that approximately one-third of the costs was borne by the patients.

**Conclusion:**

Given these circumstances, sustainable pricing and insurance coverage are necessary for both patients and facilities.

**Supplementary Information:**

The online version contains supplementary material available at 10.1007/s10147-024-02614-z.

## Introduction

Financial issues regarding the cost of fertility preservation are a major concern among adolescent and young adult (AYA) patients. In addition, post-treatment infertility and other problems associated with gonadotoxicity can lead to psychological distress and an overall decrease in quality of life. Financial concerns regarding fertility preservation influence AYA patients’ decisions and experiences [1, 2]. Along with the growing trend of fertility preservation in society, the number of pediatric and AYA patients requiring fertility preservation is also increasing [3]. Therefore, it is necessary to examine the costs associated with cryopreservation and maintenance of embryos, oocytes, ovarian tissue, and sperm, as well as the costs linked to post-fertility preservation assisted reproductive technology (ART).

In Japan, expenses for fertility preservation and subsequent ART are not covered by insurance, allowing each facility to determine its own pricing. Various types of treatment support enable pediatric and AYA patients wishing to have children in future to preserve their fertility [4–6]. In Japan, there is an urgent need to establish a system with which to explain the effects of treatment on reproductive function to AYA patients immediately following diagnosis and refer them to facilities specializing in reproductive medicine to avoid treatment delay. In such cases, the cost of reproductive medicine should not deter patients from receiving necessary fertility preservation care.

Fertility preservation is increasingly recommended for young patients whose medical diagnoses place them at risk of future infertility. AYA patients treated with cytotoxic chemotherapy or radiation are candidates for fertility preservation [5, 7, 8], as are patients with various other fertility-threatening medical conditions, such as autoimmune diseases and genetic profiles, including BRCA1 and BRCA2 [9]. In such cases, fertility preservation is recommended [3].

In the United States, fertility preservation is rarely covered by health insurance, meaning that almost all patients must undergo treatment at their own cost. For many AYA patients, it is difficult to cover these costs. Moreover, a study conducted in the United States reported that the cost of fertility preservation is an important factor in patients’ decision to avail the treatment [10]. In Israel, all AYA patients can avail fertility preservation free of charge through the Israeli National Health Insurance [10]. Assistance with the cost of fertility preservation is especially important for AYA patients, as many are still in school, dependent on family members, or unemployed because of seeking treatment. Thus, AYA patients who wish to preserve their fertility have medical needs and concerns that require special patient-centered care.

The fertility of pediatric and AYA patients may decrease or be lost due to treatment [11]. The Japan Society for Fertility Preservation, in collaboration with the Ministry of Health, Labour and Welfare’s Grant-in-Aid for Scientific Research, has leveled this field by establishing a nationwide fertility preservation network and human resource development programs related to fertility preservation, such as a certified fertility preservation navigator system and a certification system for psychologists specializing in fertility preservation [12, 13]. Financial support was initiated as a public research promotion project in April 2021. Financial support for post-fertility preservation ART was added to the project in April, 2022 [7, 13].

The aim of this study was to examine the financial burden of fertility preservation on patients and explore ways to operationalize and subsidize reproductive healthcare. We attempted to determine, through a survey, the appropriate financial support for fertility preservation therapy and ensure the continuation of this medical treatment in a sustainable manner.

## Methods

This was a cross-sectional study that utilized a mail survey. Eligibility criteria were as follows: certified facilities for fertility preservation therapy and post-fertility preservation ART. We distributed questionnaires to facilities certified by the Japan Society of Obstetrics and Gynecology and the Japanese Urological Association, following which we conducted a survey of the costs associated with each type of medical treatment. Written consent was obtained from the facility respondents. The number of subsidized cases was obtained from the Japan Oncofertility Registry (JOFR) for 2021 and 2022. To receive subsidies from local and central governments, certain conditions must be met. These conditions include the following:

1. A female patient must be under 43 years of age at the time of treatment for fertility preservation.
2. If the patient who has undergone fertility preservation is male, his wife must be under 43 years of age.
3. The patient must be diagnosed by a physician as having no or very little chance of conceiving through treatment methods other than artificial insemination, in vitro fertilization (IVF), or intracytoplasmic sperm injection (ICSI).
4. The applicant must have undergone fertility preservation treatment and conception treatment at a certified medical institution.
5. The applicant must not receive any other subsidy for the expenses for which the subsidy is sought.
6. The couple must be married or in a de facto marriage.

Responses regarding medical fees for each medical facility were mailed to the facility director and were answered by physicians, nurses, pharmacists, social workers, and administrative staff. The answers are described below:

1. Embryo freezing encompasses the costs of controlled ovarian stimulation, egg retrieval, IVF, ICSI, embryo culture, and embryo freezing.
2. Oocyte freezing includes the costs of controlled ovarian stimulation, egg retrieval, and the fee for freezing unfertilized eggs.
3. Ovarian tissue freezing includes preoperative examination fees, hospitalization charges, oophorectomy surgery fees, ovarian tissue freezing fees, and the cost of collecting and freezing unfertilized oocytes during the oophorectomy procedure.
4. Sperm freezing.
5. Testicular sperm extraction (TESE) and sperm freezing cover preoperative examination fees, hospitalization charges, intra-ovarian sperm extraction surgery fees, and sperm freezing fees.
6. ART using frozen embryos.
7. ART using frozen oocytes.
8. ART following ovarian tissue reimplantation treatment includes fees for ovarian tissue fusion, ovarian tissue reimplantation surgery, hospitalization, and ART after ovarian tissue reimplantation.
9. ART following sperm freezing.
10. Annual maintenance fees.

Graphical data were obtained using GraphPad Prism. Statistical analyses were conducted using the Student’s *t* test.

## Results

### Embryo freezing

**Fig. 1:**
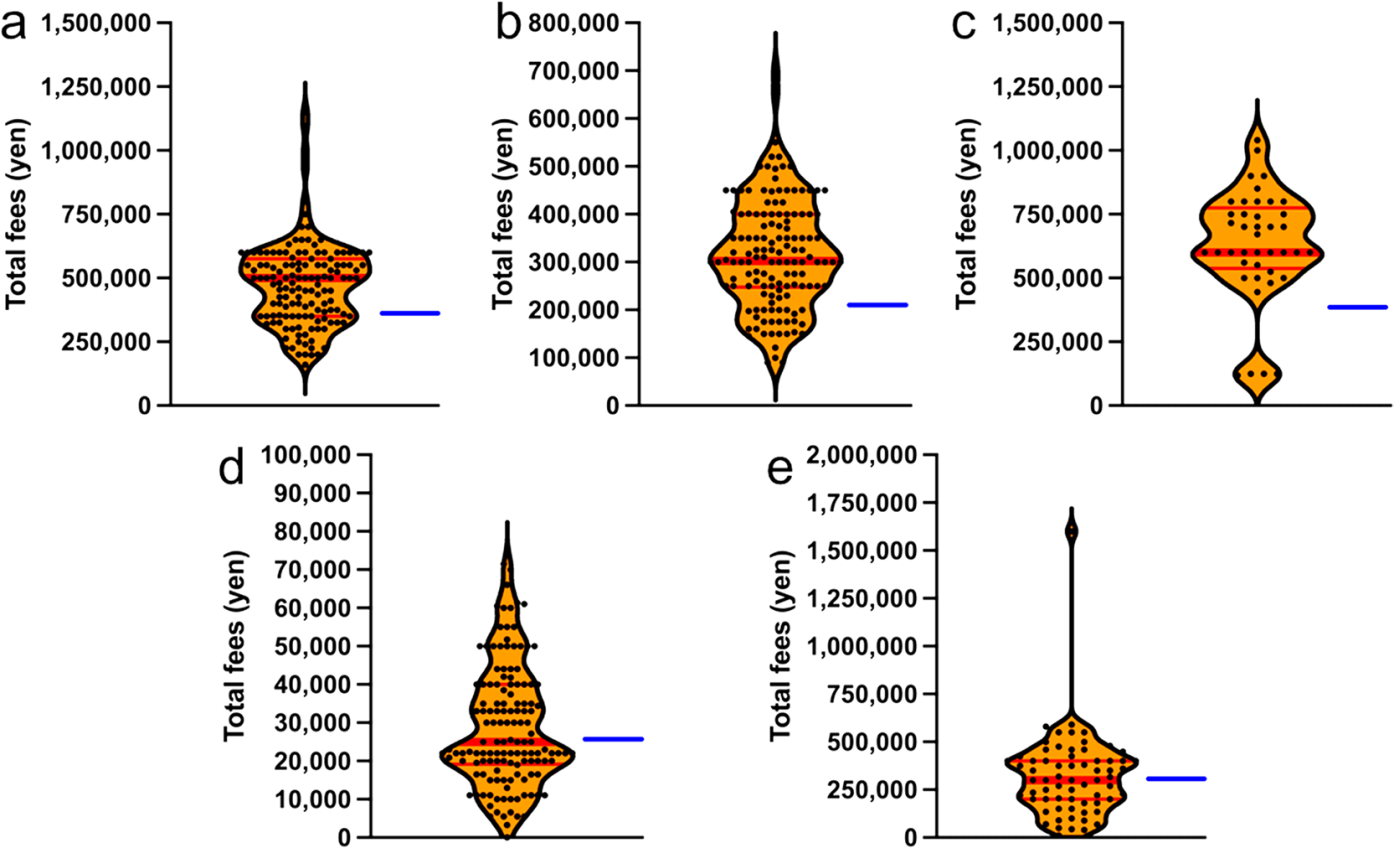
**a** Total cost of freezing embryos (controlled ovarian stimulation, oocyte retrieval, IVF, ICSI, embryo culture, and embryo freezing fees) **b** Total cost of freezing unfertilized oocytes (controlled ovarian stimulation, oocyte retrieval, and unfertilized oocyte freezing fees) **c** Total cost of freezing ovarian tissue (preoperative examination fees, hospitalization fees, oophorectomy surgery fees, ovarian tissue freezing fees, and fees for collection and freezing of unfertilized eggs during oophorectomy) **d** Cost of sperm freezing treatment **e** Total cost of sperm freezing by TESE (preoperative examination fees, hospitalization charges, TESE surgery fees, and sperm freezing fees). The dots in the graph show the average amount of payments by patients at each facility. Red line: median payment by patients. Blue line: maximum subsidy amount

The median patient payment for embryo freezing (total cost of controlled ovarian stimulation, oocyte retrieval, IVF, ICSI, culture, and embryo freezing fees) was 500,000 yen per treatment. A total of 282 treatments were subsidized by 46 prefectures in FY2021, and the total treatment cost was estimated to be approximately 141 million yen. In FY2022, 47 prefectures provided 458 subsidies, and the total treatment cost was estimated to be approximately 229 million yen. The maximum subsidy for fertilized embryo freezing-related treatment was 350,000 yen per treatment (Fig. 1a). Thus, as the median patient payment was 500,000 yen and the subsidized amount was 350,000 yen, a patient would be required to pay 150,000 yen. In examining facilities with patient payments that exceeded the 75th percentile, there was a significant range in the amount of patient payments, spanning from approximately 600,000 yen to roughly 1.2 million yen (Fig. 1a). We also divided Japan into six regional blocks and analyzed the cost of fertility preservation in each block. There were no significant differences in patient payments for the treatment of embryo freezing among regional blocks. Rather, it was found that each reproductive health facility, regardless of the regional block, set their own prices (Supplementary Fig. 1).

### Oocyte freezing

The expenses for patients who opted for oocyte freezing ranged from approximately 100, 000 yen to 800,000 yen, depending on each facility (Fig. 1b). The median payment by patients for treatment related to unfertilized oocyte freezing (inclusive of controlled ovarian stimulation, oocyte retrieval, and oocyte freezing fees) was approximately 300,000 yen per treatment. A total of 337 treatments were subsidized by 46 prefectures in FY2021, and the total treatment cost was estimated to be approximately 101 million yen for the country. In FY2022, 47 prefectures provided 537 subsidies, and the total treatment cost was estimated to be approximately 161,100,000 yen. The maximum subsidy for the treatment of frozen oocytes was 200,000 yen per treatment (Fig. 1b). Thus, as the median patient payment was 300,000 yen and the subsidized amount was 200,000 yen, a patient would be required to pay 100,000 yen.

### Ovarian tissue freezing

The median patient payment for ovarian tissue freezing (total cost of preoperative examination, hospitalization, oophorectomy, ovarian tissue freezing, oocyte collection, and freezing fees at the time of oophorectomy) was 600,000 yen each time. In FY2021, 46 prefectures subsidized treatment 46 times, and the total treatment cost was estimated to be approximately 27,600,000 yen. In FY2022, 47 prefectures provided 80 subsidies, and the total treatment cost was estimated to be approximately 48,000,000 yen. The maximum subsidy was JPY 400,000 per treatment (Fig. 1c). Thus, as the median patient payment was 600,000 yen and the subsidized amount was 400,000 yen, a patient would be required to pay 200,000 yen.

### Sperm freezing

The median patient payment for sperm freezing was approximately 25,000 yen per treatment. In FY2021, 46 prefectures subsidized 265 treatments, and the total treatment cost was estimated to be approximately 6,625,000 yen. In FY2022, 47 prefectures subsidized 472 treatments, and the total treatment cost was estimated to be approximately 11,800,000 yen. The maximum subsidy amount was 25,000,000. The maximum subsidy per treatment was 25,000 yen (Fig. 1d). Although the median payment by patients and maximum subsidy amount were consistent, the amount paid by patients increased at high-cost medical institutions.

### Testicular sperm extraction and sperm freezing

The median patient cost for treatment related to sperm freezing by TESE (inclusive of preoperative examination fees, hospitalization fees, TESE surgery fees, and sperm freezing fees) was 300,000 yen per treatment. In FY2021, 46 prefectures subsidized 5 treatments, and the total treatment cost was estimated at approximately 1,500,000 yen. In FY2022, 47 prefectures subsidized 11 treatments, and the total treatment cost was estimated to be approximately 3,300,000 yen. The maximum subsidy was 350,000 yen per treatment, indicating that many treatments were provided within the scope of the subsidy (Fig. 1e).

### ART using frozen embryos

**Fig. 2:**
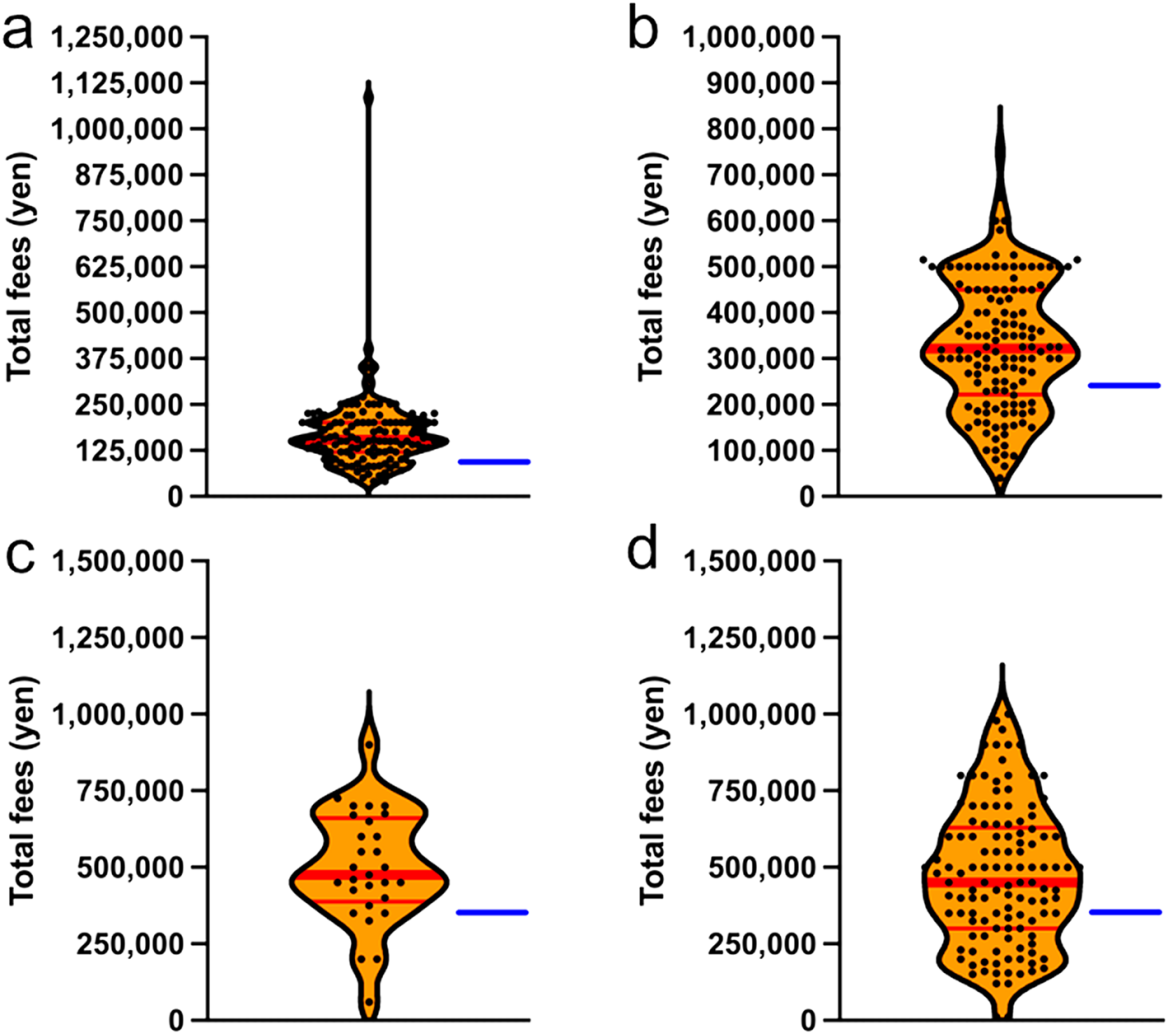
**a** Cost of assisted reproductive medical treatment using frozen fertilized embryos **b** Cost of assisted reproductive medicine using frozen unfertilized oocytes **c** Cost of assisted reproductive medical treatment after reimplantation of ovarian tissues **d** Cost of assisted reproductive medicine using frozen sperm. The dots in the graph show the average amount of patient payments at each facility. Red line: median patient payment. Blue line: maximum subsidy amount

The median patient cost for ART using frozen embryos was 150,000 yen per treatment. In FY2022, 47 prefectures provided subsidies, and approximately 13,200,000 yen was paid based on the median treatment cost. The maximum subsidy was 100,000 yen per treatment. As the median payment by patients and the maximum subsidy were 150,000 yen and 100,000 yen per treatment, respectively, approximately one-third of the cost was borne by the patients (Fig. 2a).

### ART using frozen oocytes

The median patient payment for ART using frozen oocytes was 325,000 yen per treatment. In FY2022, 47 prefectures provided 27 subsidies, and the total treatment cost was estimated to be approximately 8,910,000 yen. The maximum subsidy was 250,000 yen per treatment (Fig. 2b).

### ART after ovarian tissue reimplantation treatment

The median patient payment for assisted reproductive medical treatment after ovarian tissue reimplantation was approximately 500,000 yen per visit. However, the maximum subsidy was approximately 300,000 yen per visit, which did not cover all costs (Fig. 2c).

### ART after sperm freezing

The median payment by patients for ART using frozen sperm was approximately 500,000 yen. However, the maximum subsidy amount was approximately 300,000 yen, which did not cover all costs (Fig. 2d).

### Maintenance fees per year

**Fig. 3:**
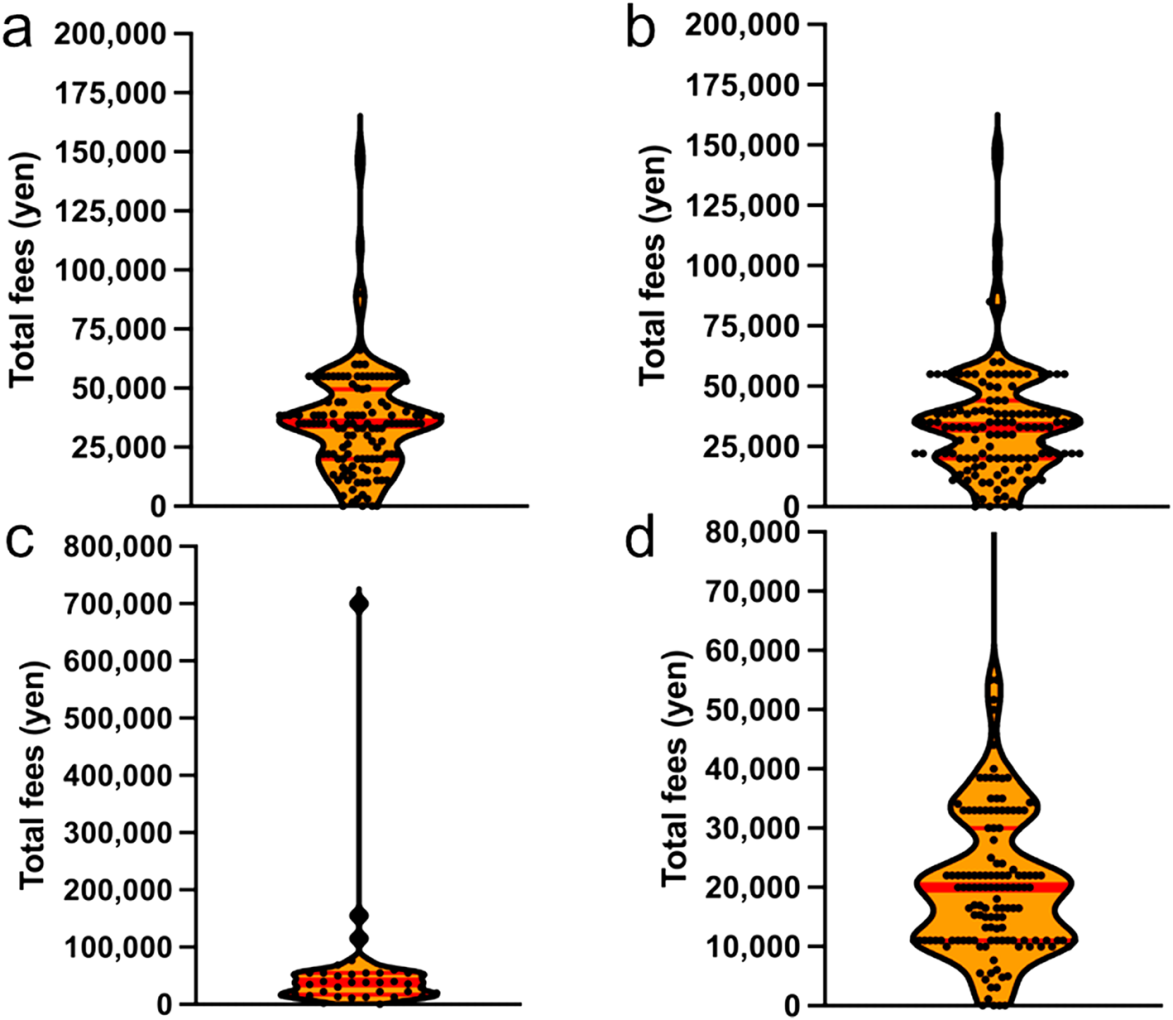
**a** Embryo cryopreservation maintenance fees for fertilized embryos **b** Cryopreservation and maintenance fees for unfertilized oocytes **c** Ovarian tissue cryopreservation and maintenance fees **d** Sperm cryopreservation and maintenance fees The dots in the graph show the average amount of patient payments at each facility. Red line: median patient payment

The median payment by patients was 35,000 yen per year for embryo maintenance (Fig. 3a), 35,000 yen per year for oocyte maintenance (Fig. 3b), 35,000 yen per year for cryopreservation of ovarian tissue (Fig. 3c), and 20,000 yen per year for sperm cryopreservation maintenance (Fig. 3d). Cryopreservation maintenance fees are not publicly subsidized in Japan, as of March 31, 2024; however, designing a future subsidy system is necessary.

## Discussion

The financial burden of fertility preservation is a major issue affecting decision-making among pediatric and AYA patients [1, 14]. In Japan, the cost of fertility preservation and subsequent ART is not covered by insurance and each facility is free to set its own prices. Several nations currently have subsidy systems for fertility preservation, with certain countries providing insurance coverage. In Europe, this trend is particularly pronounced, with the procedure being provided free of charge in countries, such as the UK, France, Denmark, Spain, and the Netherlands [15]. However, many countries in Asia, South America, and Africa have not received sufficient economic support from their respective governments. In Japan, a subsidy system has been established as part of a research project, and it is necessary to demonstrate the outcomes of fertility preservation to sustain this subsidy [7, 16]. Financial support for post-fertility preservation ART was also offered from April, 2022 in Japan [13]. The Japanese government has initiated a subsidy program as part of a research project. Indeed, financial support is necessary to enable pediatric and AYA patients to engage in treatment [12, 17].

The Japanese Ministry of Health, Labour and Welfare Foundation introduced a Cancer Survivorship Research Grant in 2017, based on a survey regarding the time and financial burden of cancer and reproductive health care for AYA cancer patients who retain the potential to have children after cancer treatment. This survey of 493 AYA cancer patients revealed that, in addition to the cost of cancer treatment, the cost of fertility preservation is an economic burden. Approximately 70% of the patients reported that their annual income was less than 4 million yen at the time of cancer diagnosis, indicating that the cost of fertility preservation alongside cancer treatment was an economic burden [18].

Conversely, however, providing patients with the option to decide whether to pursue fertility preservation or seek fertility information is crucial, not only from a medical perspective but also from a psychological one. Studies have shown that impaired fertility significantly impacts quality of life during survivorship and is associated with poorer mental health outcomes [19, 20]. Therefore, it is essential to understand the psychological needs of patients with cancer to prevent long-term distress. Reproductive-age cancer patients who face disrupted family planning due to infertility often experience heightened reproductive concerns [21, 22].

In recent years, a network for cancer and reproductive healthcare coordination has been established throughout Japan, alongside the development of a system for providing information and decision-making support to patients [13]. However, the cost of fertility preservation using ART remains heavy and is not covered by insurance. This is an urgent issue requiring resolution. In 2017, the Japan Society of Clinical Oncology published the 2017 edition of “Practice Guidelines for Fertility Preservation in Children, Adolescents, and Young Adults with Cancer,” which emphasized the need for closer collaboration between oncologists and reproductive physicians, the participation of nurses, pharmacists, psychologists, and other healthcare professionals, and strict indications for fertility preservation therapy [7, 23, 24].

A limitation of this study was the likelihood of response bias stemming from questionnaires being sent to medical facilities. Approximately 25% of the facilities failed to respond to the survey. Future research should aim to incorporate insights from nonresponsive facilities.

This study investigated the financial burden on AYA patients, exploring appropriate ways to operationalize and subsidize reproductive healthcare. In this study, we found that two-third of the cost of fertility preservation for AYA patients in Japan is publicly subsidized, while one-third is paid for by the patients. Currently, the amount paid by patients varies among reproductive care facilities. However, to ensure access to fertility preservation care for AYA patients, it is desirable to standardize the cost or introduce public insurance. We must keep track of the evolution of budgets and policies related to fertility preservation over time while also exploring the long-term reproductive and psychological advantages of fertility preservation for cancer survivors to assess its efficiency. This assessment will significantly impact the shaping of future fertility preservation policies in each nation.

## Supplementary Information

Below is the link to the electronic supplementary material.

Supplementary file1 Supplementary Fig. 1 Cost of fertility embryo freezing in six regional blocks in Japan (TIF 674 KB)

## Abbreviations

AYA: Adolescent and young adult
ART: Assisted reproductive technology
JOFR: Japan Oncofertility Registry
IVF: in vitro Fertilization
ICSI: Intracytoplasmic sperm injection
TESE: Testicular sperm extraction
