# Supplementary figures and images for: Out-of-pocket fertility preservation expenses: data from a Japanese nationwide multicenter survey
Source: Int J Clin Oncol. 2024 Sep 4;29(12):1959–66. doi: 10.1007/s10147-024-02614-z (PMC11588863; doi:10.1007/s10147-024-02614-z)

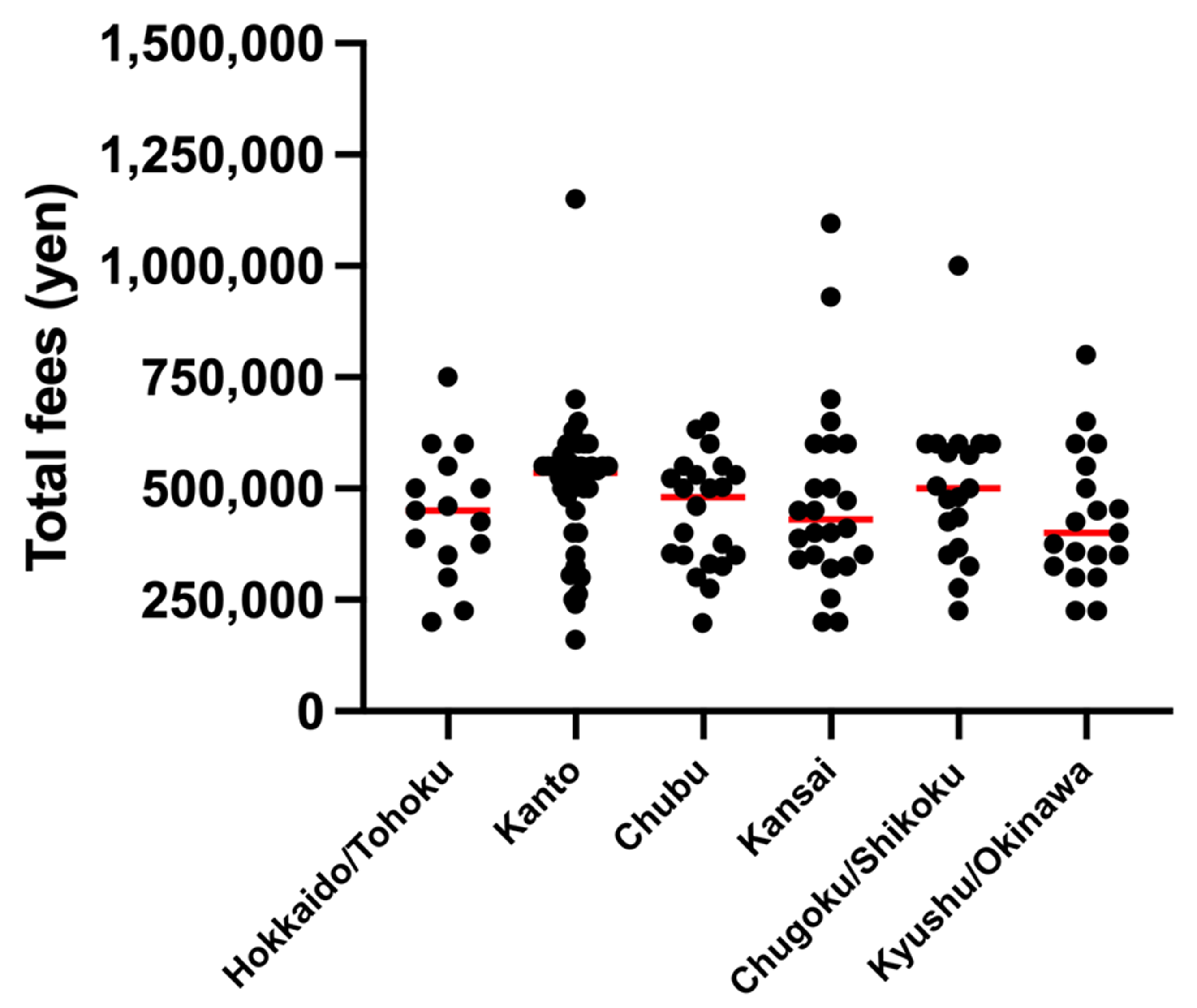

Supplement: Supplementary file 1 — Supplementary file1 Supplementary Fig. 1 Cost of fertility embryo freezing in six regional blocks in Japan (TIF 674 KB) [file 10147_2024_2614_MOESM1_ESM.tif]
